# A quality improvement project to improve handover in the integrated assessment liaison team

**DOI:** 10.1192/bjo.2021.519

**Published:** 2021-06-18

**Authors:** Jemma Hazan, Kirtana Vallabhaneni

**Affiliations:** 1 Camden and Islington Foundation NHS Trust; 2 Whittington Health NHS Trust

## Abstract

**Background:**

Efficient handovers are integral to patient care. Challenges to handover for liaison psychiatry included high patient and staff turnover and varied handover approaches across the multidisciplinary team (MDT).

**Method:**

MDT focus groups and questionnaires explored change ideas. PDSA cycles were used to design a structured handover.

We aimed to:

Reduce handover time to 30 minutes.

Improve communication using the SBAR tool.

Implement a multidisciplinary teaching schedule in the time saved.

Daily measures:

Handover timing

Team Satisfaction (Individuals ranked handover as ‘good’, ‘average’, or ‘poor’)

Weekly measures:

Semi-qualitative questionnaires triangulated areas for improvement.

Emails, posters and team meetings provided team feedback regarding QI progress.

**Result:**

A structured twice-daily handover format incorporating SBAR, allocated handover coordinators and documentation was created. Weekly MDT teaching sessions were developed.

Over 4 weeks, ‘good’ handover ratings increased from 22% to 65%; ‘poor’ ratings decreased from 25% to 8%. Mean handover time decreased from 37 minutes to 28.5.

The team viewed SBAR as a positive efficiency-promoting tool. MDT teaching improved team communication and confidence. Documentation is an area to improve.

**Conclusion:**

Structured handover has promoted efficiency and effective information-sharing amongst the liaison psychiatry team.

Interdisciplinary teaching can promote inclusive team feeling and encourage confidence across the MDT.

